# Upper Limb Home-Based Robotic Rehabilitation During COVID-19 Outbreak

**DOI:** 10.3389/frobt.2021.612834

**Published:** 2021-05-24

**Authors:** Hemanth Manjunatha, Shrey Pareek, Sri Sadhan Jujjavarapu, Mostafa Ghobadi, Thenkurussi Kesavadas, Ehsan T. Esfahani

**Affiliations:** ^1^Human in the Loop Systems Laboratory, Department of Mechanical and Aerospace Engineering, University at Buffalo, Buffalo, NY, United States; ^2^Health Care Engineering Systems Center, University of Illinois Urbana-Champaign, Champaign, IL, United States

**Keywords:** COVID-19, robotic rehabilitation, home-based monitoring, haptic, mental engagement, recovery

## Abstract

The coronavirus disease (COVID-19) outbreak requires rapid reshaping of rehabilitation services to include patients recovering from severe COVID-19 with post-intensive care syndromes, which results in physical deconditioning and cognitive impairments, patients with comorbid conditions, and other patients requiring physical therapy during the outbreak with no or limited access to hospital and rehabilitation centers. Considering the access barriers to quality rehabilitation settings and services imposed by social distancing and stay-at-home orders, these patients can be benefited from providing access to affordable and good quality care through home-based rehabilitation. The success of such treatment will depend highly on the intensity of the therapy and effort invested by the patient. Monitoring patients' compliance and designing a home-based rehabilitation that can mentally engage them are the critical elements in home-based therapy's success. Hence, we study the state-of-the-art telerehabilitation frameworks and robotic devices, and comment about a hybrid model that can use existing telerehabilitation framework and home-based robotic devices for treatment and simultaneously assess patient's progress remotely. Second, we comment on the patients' social support and engagement, which is critical for the success of telerehabilitation service. As the therapists are not physically present to guide the patients, we also discuss the adaptability requirement of home-based telerehabilitation. Finally, we suggest that the reformed rehabilitation services should consider both home-based solutions for enhancing the activities of daily living and an on-demand ambulatory rehabilitation unit for extensive training where we can monitor both cognitive and motor performance of the patients remotely.

## 1. Introduction

COVID-19 has affected numerous sectors of society, particularly healthcare workers and patients. In this regard, stroke patients are no exception, about 4 million stroke survivors live in the United States today and as many as one-half struggles with chronic motor deficits (CDC, 2017). Nearly one-third of all stroke survivors have a significant residual disability, with older individuals generally experiencing slower functional recovery (Langhorne et al., 2011). These patients face challenges in continuing their physical therapy due to access barriers to quality rehabilitation settings and services imposed by social distancing and stay at home orders due to COVID-19 outbreak.

Besides, 32% of patients recovering from COVID-19 already have comorbid conditions, such as stroke and some others suffer from post-intensive care syndrome (PICS) due to prolonged stay in ICU (Hermans and Van den Berghe, 2015; Sheehy, 2020). According to a systematic review performed on 18 Chinese studies and one Australian study, 20% of the infected patients required intensive care unit (ICU) admissions, out of which 33% suffered from acute respiratory distress syndrome and 13% suffer from acute cardiac injury (Rodriguez-Morales et al., 2020). Some of these patients show symptoms related to central and peripheral nervous system manifestations (Mao et al., 2020). Moreover, prolonged stay in ICU causes neuromuscular complications that affect limbs, respiratory muscles, and sensory nerves. These complications cause neurological impairments as well as muscular impairments, such as severe muscle weakness, reduced joint mobility, leading to the difficulties in performing activities of daily living (ADL) (Korupolu et al., 2020). These neuromuscular complications can be mitigated with the help of mobility and interventions, such as (1) passive, active-assisted, or resistive therapy; (2) repetitive therapeutic exercises; (3) functional mobility; and (4) occupational therapy for the activities of daily living (ADL) (Korupolu et al., 2020). Moreover, there is a significantly greater incidents of acute ischemic stroke in patient with COVID-19 infection compared to those without infection pointing the vulnerability of COVID-19 patients (Belani et al., 2020). Indeed, about 5% (Felten-Barentsz et al., 2020) of the admitted COVID-19 patients to the hospital may show severe symptoms and require extensive ICU stay.

However, the COVID-19 burden on the healthcare facilities worldwide is causing an early discharge of the existing patients, suspension of new patient admissions, and reduction in activities to reduce contact. For instance, in Europe alone, COVID-19 has affected access to rehabilitation services for about 2 million people (Andrenelli et al., 2020). The guideline offered by the World Health Organization for inpatient rehabilitation in COVID-19 requires daily health checks for personnel, continuous staff training on changing protocols/guidelines, use of personal protective equipment, cancellation of non-essential therapies, following proper hand hygiene instructions, and use of telecommunication for clinical interviews. Moreover, healthcare workers will be required to attend early discharged patients from acute care, decontaminate the shared equipment, prohibit group therapy, allocate a separate unit to all the patients, and provide one-on-one therapy (Bartolo et al., 2020; Sheehy, 2020). Even if inpatient rehabilitation is remodeled and available at a healthcare facility, the amount of time invested by the health care staff in practicing infection control measures decreases their work efficiency (Sheehy, 2020).

To reduce the burden on healthcare systems and provide a safe space for the patient to continue the therapy, the current rehabilitation programs should be transformed into telerehabilitation. Telerehabilitation refers to the therapy being conducted away from the hospital setting, mainly home-based or community based, which allows the users to perform a customized program of therapeutic activities. Almost, all research or review articles published in response to the physical therapy and rehabilitation needs during COVID-19 emphasize on the importance of the tele-rehabilitation and home exercise (Bettger and Resnik, 2020; Farzad et al., 2020; Zhu et al., 2020) and some even provide a guideline on how to approach staff training, patients evaluations, and discharge in such settings (Rosen et al., 2020). In this review article, we propose a hybrid model incorporating home-based telerehabilitation and inpatient treatments through ambulatory robotic rehabilitation services as a more effective solution during COVID-19 and similar pandemic that may accrue in future.

**Figure 1:**
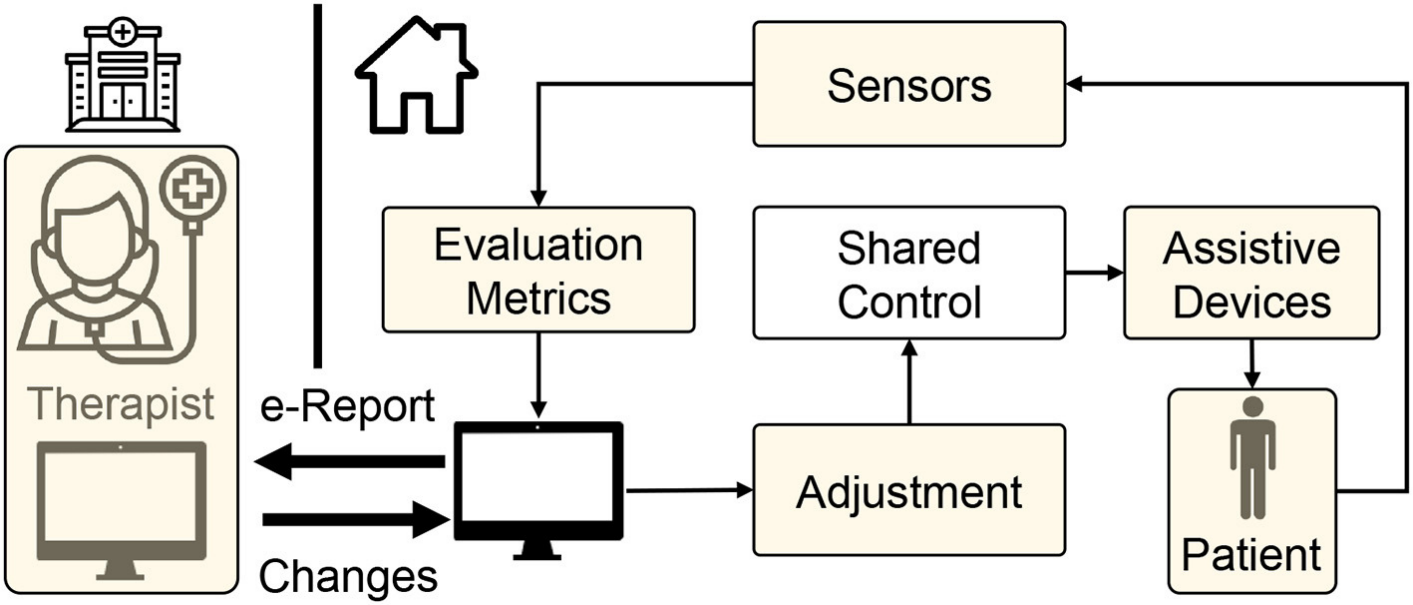
Schematic of tele-rehabilitation where patients can continue their rehabilitation with the help of an assistive device while therapist can monitor the progress remotely.

In telerehabilitation, an occupational therapist or a healthcare provider works closely with the patient and provides feedback and instructions through web interfaces. By monitoring the progress of the patient, they can also make necessary changes to the exercise regime. However, the therapists might not have enough time to monitor the patient's progress online due to the increased COVID hospitalizations. Nonetheless, thanks to the technological advancements in the last two decades, considerable effort has undergone toward building new physical platforms, such as robotic and orthotic systems (Brennan et al., 2009; Housley et al., 2018) to facilitate the telerehabilitation process and also improve the outcome of motor function recovery (Figure 1). In particular, using robots and orthotics equipped with haptic feedback or haptic assistance is viewed as an alternative solution to physical therapy (Krebs and Hogan, 2012; Linder et al., 2013). These systems can be effectively used to continue the rehabilitation procedure even during the COVID-19 pandemic in-home and community centers. For patients who face difficulties due to traveling disabilities or limited transportation (Holden, 2005), community-based rehabilitation can be extended to ambulatory robotic rehabilitation services.

Substituting the physical therapy with telerehabilitation approach requires four key components (Figure 1). First, *delivering assistance:* Since the therapists are not present to guide the patient physically, there is a need for low-cost devices that can provide necessary support (Frolov et al., 2018). In this regard, as discussed previously, haptic devices and robotic systems offer a promising solution. Second, *enhancing engagement and social support:* Even with repetitive support from robotic systems, the rehabilitation outcome may not be superior to physical therapy without patient's engagement (Blank et al., 2014). So, encouraging and maintaining patient's engagement in telerehabilitation is of paramount importance. Third, *assessing the progress:* As patients cannot access the hospital facilities frequently during COVID-19 restrictions, periodic assessment of functional status is impeded; thus, there is a requirement for remote assessment devices and metrics (Nordin et al., 2014; Frolov et al., 2018). Consequently, the telerehabilitation approach should support a wide array of low-cost sensors through which the therapist can assess the patient's recovery. Finally, *adaptation:* As the patient's needs vary throughout the rehabilitation regime, robotic/haptic systems' ability to adapt plays a vital role in delivering necessary rehabilitation assistance while adhering to social distancing norms during COVID-19 outbreak. With this backdrop, in the succeeding sections, we provide brief literature in these four critical areas in the context of upper limb rehabilitation.

In the subsequent sections, we will review the main components of telerehabilitation (home or community based) related to *delivering assistance, enhancing engagement and social support, assessing the progress*, and *providing adaptation* to provide successful therapy during COVID-19 outbreak.

## 2. Assistance Delivery

In the current pandemic, home environment serves as the best solution to deliver remote rehabilitation to patients. It reduces the burden on inpatient services and, at the same time, prevents the spread of disease to the patient. The main objectives of delivering therapy in such settings are to (1) facilitate repetitive task training with real-time feedback about performance, and (2) maintain high patient engagement during training (French et al., 2016).

### 2.1. Tele-Rehabilitation Framework

Tele-rehabilitation was first documented in 1959 when an interactive video was first used at the Nebraska Psychiatric Institute to deliver mental health services. With the advent of the Internet and the availability of large medical records, telerehabilitation/telemedicine received more attention in mid-1990s focusing on the proof of concept with few clinical trials. Since the early 2000, there has been a surge of tele-rehabilitation mainly focused on rural areas. By 2016, around 125,000 stroke patients were reported to have used telerehabilitation for treatment (Peretti et al., 2017). For years, researchers and practitioners utilized telerehabilitation to reduce inpatient hospitalization duration and reduce the cost of rehabilitation for patients. Cramer et al. (2019) has shown that the efficacy of upper limb home-based telerehabilitation is comparable to the therapy delivered in clinical settings. Many ADL skills, such as using a fork and spoon, twisting doorknobs, and being able to manipulate simple objects, require fine motor control of the patient's hand and are better suited for home-based therapy.

Rehabilitation therapy also requires the patient to perform high-intensity exercises and get periodic assessment from a therapist, which is not generally feasible in home environments due to the lack of equipment, thus, home-based rehabilitation should be combined with outpatient rehabilitation services offered by rehabilitation clinics and community rehabilitation centers. Ru et al. (2017) and Dean et al. (2018) have recently shown that patients participating in community-based rehabilitation programs, when coupled with home-based exercises, demonstrated enhanced motor function, daily activity, and social activity. Community rehabilitation centers or kiosks mentioned in the above studies use a video/audio communication channel to connect the therapist to the patient and allow a continuous exchange of information (Figure 1). Patients perform the physical exercise while being remotely monitored and assessed by a physiotherapist via video-conferencing. Such telerehabilitation services provide a cost-effective solution to deliver and monitor long-term therapeutic interventions. In this context, Holden et al. (2007) developed a telerehabilitation system that provides real-time interaction between a patient at home and a therapist located at a clinic. Reinkensmeyer et al. (2002) developed a web-based telerehabilitation system for the patient to practice simple movements using an adaptive joystick with force feedback. The therapist can track improvements in training. Another low-cost telerehabilitation platform is Habilis (Motus, 2020) developed for the Clinical Leading Environment for Assessment and Validation of Rehabilitation Protocols for Home Care (CLEAR) project under the European Union. At home, these telerehabilitation services can be accessed via mobile phones or tablets connected to the Internet. These technological devices provide an affordable solution to connect and directly interact with sensors (Ameer and Ali, 2017). Such devices also enable offline use of services, such as pre-recorded sessions by therapists and online services, such as video-conferencing. In the absence of such services, patients can follow some home exercise guides, such as one prepared by Ambrose et al. (2020).

The development of telerehabilitation requires a reliable communication network and tailored software systems to deliver rehabilitation support effectively. In this regard, Hosseiniravandi et al. (2020) provide a scoping review of different software systems designed to address the delivery problems of home-based telerehabilitation. The review included systems with various functional features, such as exercise plan management, report generation, and task scheduling. On similar lines, Fiani et al. (2020) provide a review on the development, usage, and technological advances of telerehabilitation. The authors also provide suggestions on advancements of telerehabilitation during COVID-19. Additionally, the development of an effective telerehabilitation service requires identifying methods and material to evaluate patients' existing functional status such that the intensity of exercise can be modulated. The service should efficiently collect and document patient data to monitor exercise intensity and patients' progress during therapy. Tele-rehabilitation platforms, such as VidyoHealth™and Habilis™enable synchronous and asynchronous data collection. These services enable setting up automatic training schedules, recording patients' activity, evaluating their functional status, and manipulating the factors to vary the intensity of therapy based on their progress (Middleton et al., 2020).

In the remainder of this section, we will focus on the main components of telerehabilitation necessary to assist, evaluate the patient's state, assess the patient's engagement and compliance, and suggest adaptation based on the patient's functional status.

### 2.2. Robotic Devices

Robotic rehabilitation has shown promising results in lab environments. During clinical trials, their validation demonstrated huge potential in patients' recovery (Maciejasz et al., 2014) and can be used as an alternative to physical therapy. These robots sense the user's movement and use that information to provide force feedback or plan subsequent motions. The robot can interact with the patients in three possible ways: (1) passive (patient-driven), (2) active (robot drives), and (3) challenge (resist the forces applied by patients). In this regard, Frolov et al. (2018) provide a scoping review of different robotic devices used in rehabilitation. Even though much robotic rehabilitation systems are in use, only a few robots have been developed for home-based telerehabilitation. For instance, only robots, such as Hand Mentor, Foot Mentor (Motus Nova, 2020), and SCRIPT (Ates et al., 2017) have been successfully used in the home setting. The Hand and Foot Mentor devices provide active assistance to increase the range of motion in patients who have residual upper and lower extremity impairments. The patient completes a game-like training where the difficulty is modified depending on the progress. The device provides audio and video feedback along with remote monitoring through the clinician dashboard. Unlike Hand and Foot Mentor, SCRIPT provides passive assistance for finger and extension. This decreases the cost of deployment and simplifies the software algorithm design. Similar to Hand and Foot Mentor, SCRIPT provides an interactive game-like interface. To expand further, Brewer et al. (2007); Housley et al. (2018) provide a review of different telerehabilitation robotic (TRR) approaches and clinical outcomes in home-based settings. The review covers topics, such as ease of deployment, cost-effectiveness, involvement from the patients, intervention protocol, and dosing. The review concludes that future TRR design should consider the cost analysis for wide adaptation of TRRs in home-based settings.

However, most robotic rehabilitation setups are too expensive and require monitoring by a skilled operator, and are most suited for community-based rehabilitation centers and not home-based settings. In the last two decades, new low-cost haptic systems (e.g., Novint Falcon, 3dsystems Phantom, Quanzer Pantograph, and so on) have emerged and adopted for home-based rehabilitation. These haptic systems sense the user's movements and use them to assist subsequent motions by providing force feedback. Such continuous feedback is shown to enhance the rhythmic motor control by reducing the temporal variability in repeated movements (Ankarali et al., 2014). Thus, low-cost, ease of use, and low-maintenance haptic devices have attracted a lot of attention for home-based rehabilitation (Oblak et al., 2010; Piggott et al., 2016).

In addition to hardware, many researchers have studied how different force feedback strategies elicit better rehabilitation outcomes. The two most popular force feedback strategies are (1) *error-reduction (ER) strategy*, which decreases the performance error by providing active assistance to enable the patient to perform the rehabilitation tasks better; (2) *error-augmentation (EA) strategy* that increases the task difficulty to evoke a higher voluntary involvement of the patient to accomplish the goal (Israely and Carmeli, 2016). In a scoping review by Li et al. (2018) on the effect of EA and ER strategies on upper limb post-recovery showed that subjects under EA showed statistically significant motor performance improvement compared to the ER. In fact, the EA strategy aligns with the motor adaption principle, which suggests that kinematic errors generate neural signals that drive motor adaptation during movement (Schmidt et al., 2018). Even though EA and ER are widely used strategies, such therapy's outcomes will not be superior to manual therapy if the patient is not actively engaged in the therapy (Takeuchi and Izumi, 2013; Blank et al., 2014). Consequently, maintaining patients' engagement through virtual reality (VR) or augmented reality (AR) has gained significant traction.

### 2.3. Virtual Reality

**Figure 2:**
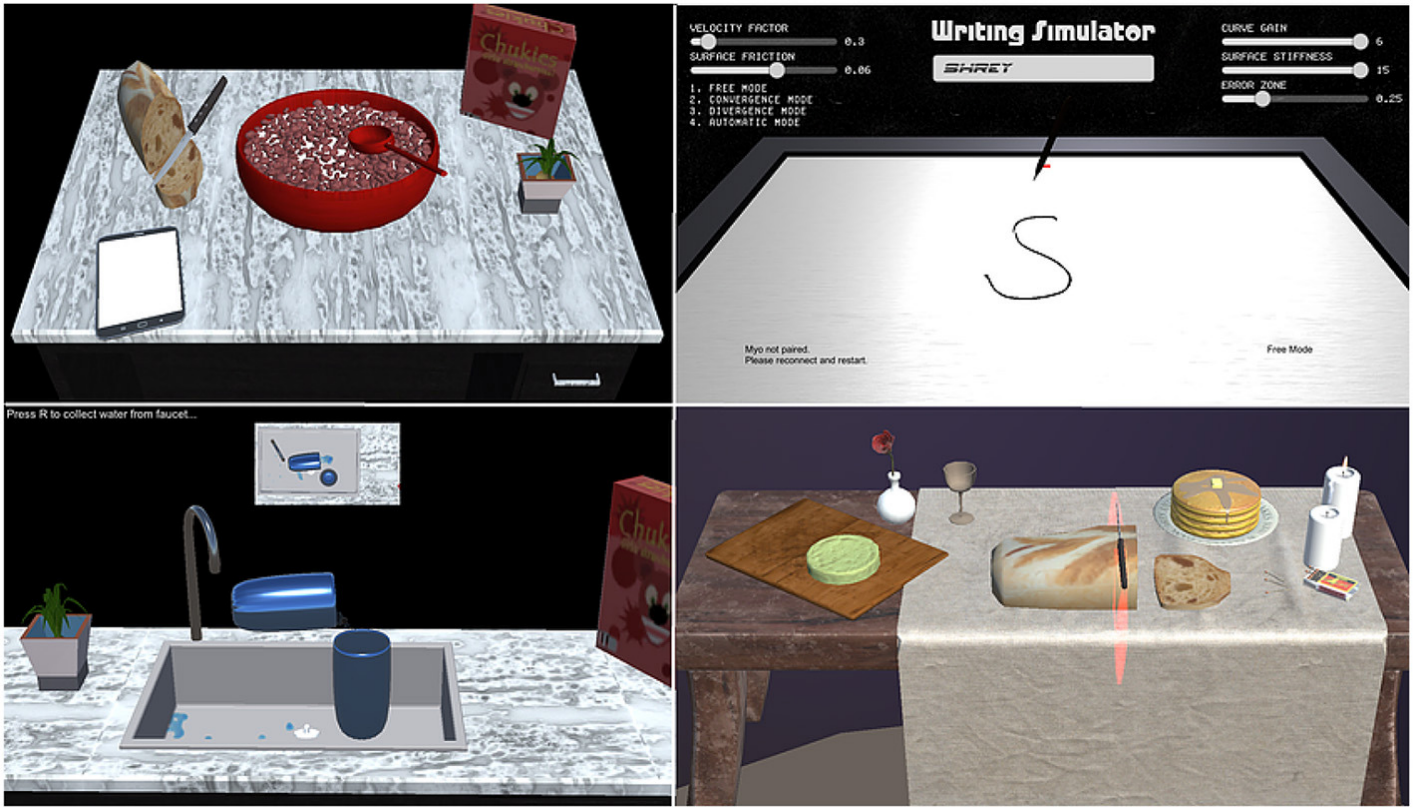
Different virtual reality (VR) games that can emulate Activities of Daily Living (ADL), such as using a spoon (eating), pen (writing), knife (cutting), and glass (pouring) in clockwise order from top-left.

VR in rehabilitation is explored as a modality to provide feedback and engage patients through immersive environments. VR refers to an artificial environment experienced through sensory stimuli (as sights and sounds) provided by a computer, and the user's actions partially determine what happens in the environment. In other words, any simulation on a computer screen may be considered VR (Figure 2). These systems cannot provide assistance/resistance to patient's movements and require a robotic or haptic system.

**Figure 3:**
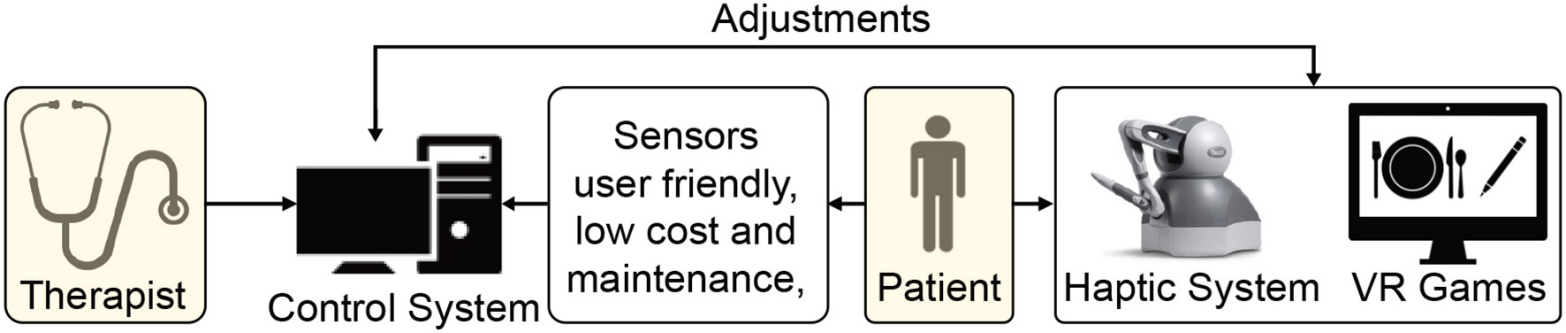
Schematic demonstrating a haptic device and virtual reality (VR)-based home rehabilitation setup.

VR offers the capability of showing the trajectory of the patient's limb movements in real-time that enhances motor learning during rehabilitation (Pareek, 2020). Moreover, tasks designed using VR can be customized to patient's needs at different therapy levels, i.e., therapists can make the task easier or challenging according to the recovery status (Figure 3). Rose et al. (2018) provides a review on VR applications in rehabilitation aiming at (1) how VR is beneficial in the health outcomes, (2) how VR can influence the patients to adhere to the rehabilitation plans, and (3) influence of haptic feedback on the performance of an individual in the VR.

Display screens have been used for a long time to present virtual environments during rehabilitation. In recent years, head-mounted VR devices have attracted a lot of attention in rehabilitation. Commercially available VR headsets (Oculus Rift^®^, Microsoft HoloLens^®^, HTC Vive^®^, and the Samsung Gear VR^®^) have added additional dimensions and intuitiveness to the VR technology (Webster and Celik, 2014). Haptic interfaces can augment the virtual interaction forces in the real world and thus complement VR with force feedback during the therapy. Moreover, tasks designed using VR can be customized to the patient's needs to adaptively challenge them according to their progress and engage them in the therapy. Emerging companies, such as Neuro Rehab VR and Peili Vision have already developed VR stroke rehabilitation systems. These systems aim to increase patient engagement by making physical therapy more enjoyable. However, these systems currently rely on commercially available gaming hardware that is not tailored for stroke patients, which limit their practical use but promise a reliable framework for home-based therapy.

## 3. Engagement and Social Support

Maintaining motivation to adhere to the therapy is challenging for the patient during unsupervised therapy in home-based and community-based settings. A combination of VR and robotic systems can provide the necessary motivation by making the exercises more comfortable, less dangerous, engaging, and entertaining. Such customizability allows the therapist to make high-intensity and repetitive training exercises more motivating, engaging, and enjoyable for the patients (Rose et al., 2018). Specifically, VR-based therapy can increase patients' engagement by creating interactive and competitive tasks that provide frequent performance feedback during the exercise (Zimmerli et al., 2013). In addition to the VR visual feedback, multimodal feedback, such as auditory and haptics can enhance the patient's engagement during the exercise. One of the promising modalities is brain–computer interfaces (BCIs).

BCIs have proved to be a useful tool in evaluating patient's engagement during therapy. They can be used to objectively assess task performance, engagement, and voluntariness (Sullivan et al., 2017; Likitlersuang et al., 2018; Manjunatha et al., 2020). Concretely, BCIs have attracted a lot of attention in quantifying mental engagement as it directly measures the subject's cognition during rehabilitation (Berger et al., 2019). Such measures can adaptively change the robot/haptic parameters to desired levels (Bartur et al., 2017). The expense and setup procedure of BCIs makes it challenging to be used in home-based environments. These sensors can be used in clinics or community-based setting where the patient's cognitive state can be monitored during his/her visit.

Another factor that impacts patients' participation in therapy is social support. Patients suffering from stroke or COVID-19 develop anxiety, depression, fatigue, and post-traumatic stress disorder. In addition to the physical or cognitive state, psychological health acts as an indicator of the surviving population's quality of living. For instance, a scoping review by Essery et al. (2017) revealed that social support is a strong predictor of home-exercise adherence. Along with social support, other main factors included self-motivation, intention, self-efficacy, and previous adherence. The study showed that social support would increase adherence by providing encouragement, boosting self-esteem, and buffering stress due to illness. Thus, as the patients are motivated to adhere to an exercise regime, the recovery is accelerated. The positive influence of social support on the outcome of patient's recovery is also in-line with previous adherence studies (DiMatteo, 2004; Jack et al., 2010).

Physical therapy and rehabilitation can improve neuromuscular functionality; however, the methods to prevent or treat depression or cognitive impairment are still lacking. Cognitive evaluation and behavioral therapy are slightly useful in improving the psychological and cognitive state. A more practical solution for enhancing psychological health is to provide motivation and emotional support to decrease their loneliness and coach them to compensate for diminished skills or lacking abilities.

In home-based therapy, family members are the primary caregivers who can provide social and moral support to the patient throughout the recovery process. Proffitt et al. (2011) indicated that activities incorporating family members might facilitate compliance and reduce patients' social isolation. The therapist can also provide additional social assistance through video-conferencing, virtual avatars (Borghese et al., 2013) designed in VR, indulging and entertaining VR games, and socially assistive robots (SAR).

### 3.1. Socially Assistive Robots

A social companion robot is defined as a robot that can assist humans in daily activities at home, workplace, and other environments (Dario et al., 2011) and possesses the skills to interact with the people socially. Social companion robots or SARs can benefit the elderly population, individuals with physical, neurocognitive impairments, and individuals suffering from depression (Lorenz et al., 2016). SAR provides a stimulating or motivating influence on individuals and reduces their loneliness. One of the main challenges of rehabilitation during COVID-19 is contact. In this regard, SAR creates a bridge between contact-based rehabilitation robotics and non-contact functionalities of the companion robotics. Therefore, SAR enables contact-free monitoring, coaching, and encouragement while also providing detailed assessments of the patient's progress.

Some popular SARs that fulfill the role of a pet are Paro (Shibata et al., 2001), NeCoRo (Libin and Libin, 2004), and Huggable (Stiehl et al., 2005). Similarly, SARs made for elderly care are Care-O-Bot (Graf et al., 2009), MobiNa, Hector (Schroeter et al., 2013), and Hobbit (Fischinger et al., 2016). These robots enable the independent living of the older population by helping them with household tasks. In addition to monitoring patients' progress and motivating them, SAR (Eriksson et al., 2005) and Clara (Kang et al., 2005) can help in rehabilitation. For example, Bandit (Eriksson et al., 2005) is a hands-off therapist robot that can navigate autonomously, demonstrate the task, monitor patients' arm activity, and remind them to follow a rehabilitation program. Clara (Kang et al., 2005) is another hands-off therapist robot that can assist patients in repetitive spirometry exercises; thus, it can be very useful for patients recovering from Acute Respiratory Distress Syndrome (ARDS).

The major challenge of SARs is to identify the social abilities from human and implementing them (Lorenz et al., 2013). SARs have to be adaptive as the interaction with a non-adaptive robot cannot result in movement synchronization (Lorenz et al., 2013). Synchronous behavior between the patient and a robot is essential for the emergence of compassion and positive emotions (Lorenz et al., 2016). In this context, Bethel and Murphy (2010) provided some measures to evaluate a robotic system in terms of interaction.

## 4. Progress Assessment

While delivering remote rehabilitation, the therapist needs to monitor the functional progress of a patient to vary the intensity to the desired level. Sarfo et al. (2018) reviewed the commonly used metrics to monitor patients' progress during telerehabilitation interventions, of which *ABILHAND, Ashworth scale, Action Research Arm Test (ARAT), Fugl-Meyer Motor scale for upper extremity (FMA-UE), Grip strength, Nine-Hole Peg test (9-HPT), Shoulder strength*, and *Wolf motor function test (WMFT*) are used to assess upper limb functionality. ABILHAND is a subjective measure of the ability to manage activities of daily living. Ashworth scale is a subjective score ranging from 0 to 4 based on the resistance to passive movement about a joint. ARAT requires a kit to test the grasp, grip, and pinch functionalities along with the gross movement capability of the upper limb. FMA-UE provides a quantitative measure for a range of functionalities involving the upper extremity, wrist, hand, coordination, speed, sensation, passive joint motion, and joint pain. 9-HPT is a standardized quantitative assessment to measure finger dexterity and requires a wooden board with nine holes and nine pegs. WMFT is a quantitative measure to assess time, functional ability, and upper extremity motor ability strength. These metrics have been extensively used for the remote assessment of upper limb functionality in chronic stroke and neuromuscular disorders, and can also provide a quantitative prior for assessing COVID-19 patients during telerehabilitation. In addition to these metrics, patients' satisfaction and cognitive state should be examined to assess both mental and cognitive engagement as they are vital aspects in the success of remote rehabilitation (Pareek and Kesavadas, 2019).

In the absence of a therapist, sensors should measure and quantify patients' exercise in the home environment. Wearable sensors have been utilized to measure and assess a wide range of motor behaviors, such as fall detection, mobility characterization, and activity recognition. Moreover, the information from these sensors can act as biofeedback in automated training. The most common information used for assessing the upper limb functionality is the trajectory of the upper limb tracked by the robot's sensors. Moreover, inertial measurement units (IMUs) provide a portable and low-cost solution to physical activity detection (Wittmann et al., 2016). IMUs placed on the upper limb can be used to monitor movements during therapy, and when placed near the ankle, can be used to characterize patients' gait. On the other hand, IMUs in mobile phones provide low-cost alternatives to external IMUs. Force-sensitive sensors can be incorporated in wearable gloves (Polygerinos et al., 2015) and fabrics to detect grasp pressure during upper extremity exercises. Moreover, force-sensitive sensors incorporated in foot-ware can measure ground reaction forces and provide better fall detection when used in combination with IMUs. IMUs and force sensors can be easily incorporated into home-based rehabilitation to detect voluntary forces from the patient. Moreover, the trajectories obtained from the haptic and VR systems are useful in tracking patients' progress.

Non-invasive physiological sensors, such as surface electromyography (sEMG) can also be used to assess changes in neuro-motor control during robotic intervention (Clark et al., 2010). A combination of sEMG sensors and IMUs has been used to monitor movement quality while assessing patients' muscle activity (Pareek et al., 2019). Such sensors provide a low-cost solution for differentiating voluntary contractions from spastic and enable automatic detection of functional ADLs, such as feeding, grooming, dressing, transferring, locomotion, and toileting in home-based therapy (Porciuncula et al., 2018). Additional physiological sensors used during therapy can record body temperature, respiratory rate, pulse rate, blood pressure, muscle activity, cognitive state, and so on (Chen et al., 2019). While these additional sensing technologies may seem redundant for the home-based setting, they may be used in the community-based rehabilitation center to provide additional insight into the patient's cognition.

New studies provide empirical evidence that closed-loop sensorimotor systems that use brain activity and haptics in robotic therapy improve the rehabilitation of upper limb (Frolov et al., 2018). Non-invasive BCIs introduce EEG signals as potential feedback capable of indicating the subject's intentions and providing his/her sophisticated cognitive state, such as the level of engagement. Popular metrics include event-related synchronization or desynchronization (ERS/ERD) (Jochumsen et al., 2013) and sensory-motor rhythms (SMR). For instance, Soekadar et al. (2015) suggested SMR as an ideal candidate for non-invasive BCI-training in stroke neuro-rehabilitation. This is because SMR is closely related to motor activities, accessible through EEG signals, and has a high signal-to-noise ratio (Soekadar et al., 2015). Moreover, studying motor learning after stroke is also possible with motor imagery measures in a passive setting (Meyer et al., 2012).

While the use of BCI in current clinical practice is viable, the remote operations may seem impractical due to setup and calibration requirements. The future generation of remote rehabilitation system can potentially use them as an alternative to traditional feedback in active rehabilitative platforms (Bamdad et al., 2015). In this regard, van Dokkum et al. (2015) conducted a literature review on different aspects of BCI application for neuro-rehabilitation. The study considered current methods useful for three applications: (1) providing feedback to adjust training tasks, (2) quantifying and measuring motor improvements, and (3) stimulating patients to encourage and make them optimize and correct themselves to execute their tasks. The authors recommended using BCI for motor rehabilitation purposes according to its adaptability to a large population and, at the same time, consider it necessary to study for more clinical results based on controlled designs to validate the impact of BCI on motor and functional recovery.

## 5. Adaptive Rehabilitation

**Figure 4:**
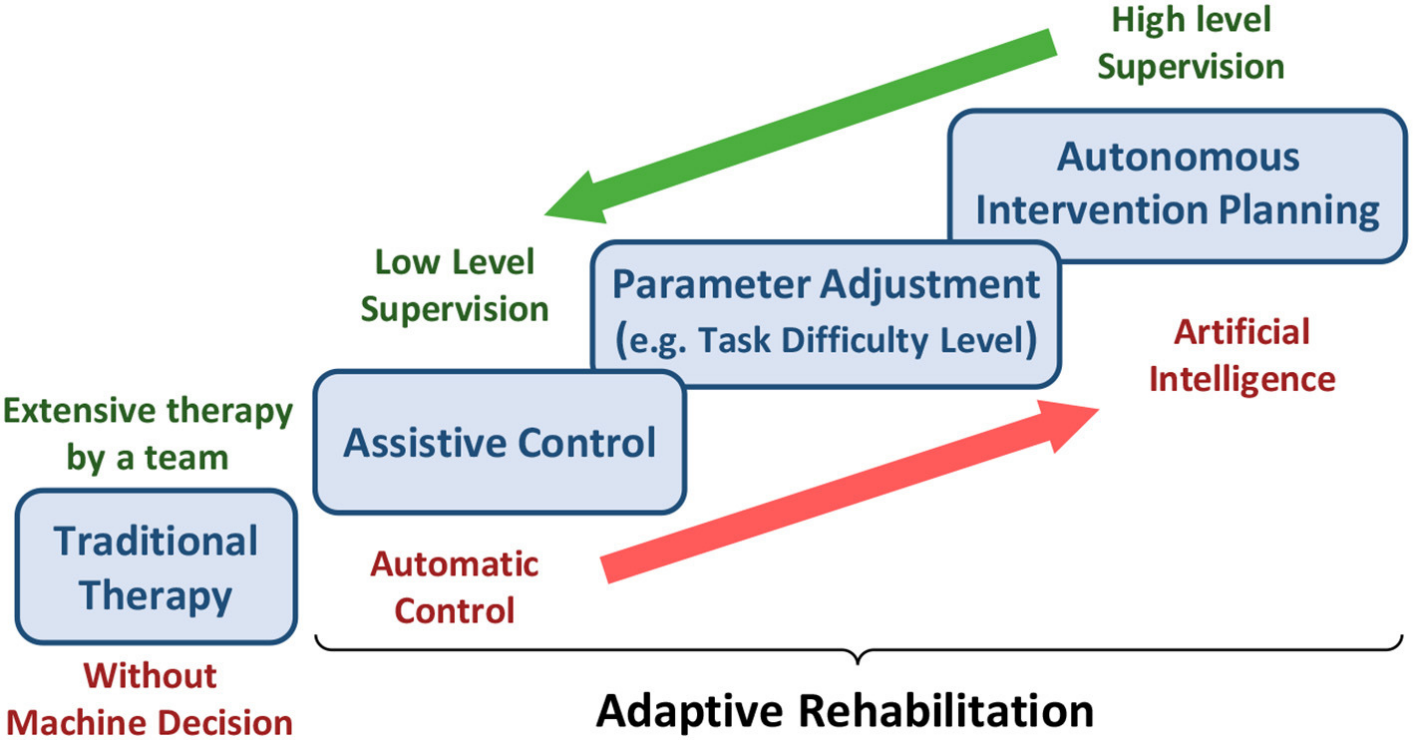
The burden of assistance and decision-making shared between experts and machines from traditional therapy to adaptive rehabilitation.

Due to the COVID-19 outbreak, the patient's rehabilitation should be shifted to a teleoperated home-based or community-based approach to reduce the therapists and inpatient facilities' burden. However, for such an approach, one of the major priorities is to devise a reliable decision-making algorithm as an alternative to the therapist (Figure 4). Such a decision-making algorithm must determine when, how, and to what degree the interventions must be modified and adapt accordingly to improve the patient's functional recovery. The adaptation should be based on the patients' existing state and recovery progress. For inferring the patient's state, physiological signals are an indispensable modality. For example, the physiological signals, such as EEG, EMG, and eye tracking can be used passively to understand the state of the patients and their level of engagement. They can also actively modify the rehabilitation parameters (e.g., assistance level provided by haptics/robotics system or VR game difficulty). Some modalities cannot be obtained in home-based settings as they desire low-cost sensors with a minimal setup procedure. However, various sensors ranging from EEG to IMUs can be used in the community or ambulatory rehabilitation. In this regard, significant measures have been adopted by researchers to implement adaptive rehabilitation services where VR and haptic devices can be adapted using physiological signals.

### 5.1. Adaptation of Virtual Reality Interfaces

VR has facilitated the implementation of adaptive rehabilitation approaches for two reasons. First, the relative ease and flexibility in developing VR environments compared to building physical interfaces in the real world. The VR systems can be easily adapted to both home, community, and ambulatory rehabilitation. Second, the patient's performance and progress can be measured easily with respect to the accomplishment of a mission through a series of tasks or games. Moreover, being involved in a game or even serious virtual tasks through an interactive environment can significantly increase cognitive engagement, which is traditionally provided by social communication between a patient and the rehabilitation team members.

In VR, the game difficulty can be easily adjusted depending on the patient's engagement and progress. In this category, Nirme et al. (2011) has designed a Rehabilitation Game System (RGS) based on VR. They developed two algorithms capable of controlling the task difficulty that an RGS user is exposed to and provide controlled variation in the therapy. In this regard, Hocine et al. (2015) have also recently studied related works in the adaptive adjustment of task difficulty. They hypothesized that a dynamic adaptation of task difficulty based on the subject's abilities and performance surpasses the other two methods reported in previous works, which make either an incremental or a random change in the task's difficulty level (Cameirao et al., 2010; Rabin et al., 2011). The results of their study demonstrated that the stroke patients under experiments with dynamic adaptation methods gained a higher amplitude of movement, which is considered a positive sign of recovery (Hocine et al., 2015).

An interesting result of the scoping review done by Bamdad et al. (2015) is that the work based on VR control (VRC) dedicates half of the research papers in BCI-based rehabilitation. Barzilay and Wolf (2013) have recently proposed an effective VR framework to improve triceps performance by designing a set of adaptive rehabilitation games that work with respect to some biofeedbacks. They provide these biofeedbacks through a learning system that estimates the biological model from raw data being acquired from hand motion and muscle activities. In this regard, Pirovano et al. (2012) have also developed a framework of self-adaptive games for rehabilitation at home. In such a framework, they have considered the game design to be (1) capable of being integrated into general-purpose rehabilitation stations, (2) consistent with the constraints posed by the clinical protocols, (3) inclusive of both effective and functional movements to reach the rehabilitation goals, and (4) adaptive to the patient's current status and his/her estimated progress. They utilized a fuzzy system to monitor the execution of exercises and a Bayesian adaptive approach to modify the gameplay with respect to the current performance and estimated progress of the patient as well as the exercise plan that is each time instructed by the therapist (Pirovano et al., 2012). This adaptive game engine is extended in a more recent research conducted by Pirovano et al. (2016), where they have also addressed how the adaptation of task difficulty can be performed with respect to the patient's performance as well as real therapist inputs to increase the level of engagement. To this end, a virtual therapist (Borghese et al., 2013) guarantees the patient to be properly challenged and, at the same time, motivated, safe, and supervised. Pirovano et al. (2016) also introduced a more independent autonomous rehabilitation game engine that provides a home-based framework needless of close supervision by a therapist. In a recent overview, Vaughan et al. (2016) presented state-of-the-art self-adaptive technologies within VR training.

Despite the recent advances in VR, its feasibility in the clinical rehabilitation setting is limited in terms of application, education, and research (Laver et al., 2011). Although many studies aiming at the development and evaluation of VR-based rehabilitation systems exist, very few have been evaluated outside laboratory settings. Three major limitations have been reported for the use of VR in rehabilitation, latency between input and output devices, underestimation of perceived distance in real world, and motion sickness (Morel et al., 2015). Latency is the delay between patient's action using input device and its corresponding reaction using output device in the virtual environment. Latency affects rehabilitation efficacy by delaying the timing of stimulus presented to the patient. Improper relation between the perceived distance in real and virtual environments, motion sickness, eye fatigue, headaches, nausea, and sweating caused due to prolonged exposure to head mounted displays limit the efficacy of VR systems (Laver et al., 2011; Yates et al., 2016; Park et al., 2019). Additionally, the cost of VR development, aggravated by the poor reception of these technologies by older stroke patients, inhibits these systems' feasibility. However, these limitations should be evaluated using studies with larger sample sizes and post-intervention follow-up measures (Yates et al., 2016).

### 5.2. Adaptation of Haptic/Robotic System

The success of rehabilitation robots in physical therapy encourages the researchers to develop an adaptive robotic rehabilitation strategy (Mounis et al., 2019). For instance, Kan et al. (2011) proposed an automated rehabilitation robotic system that guides stroke patients through an upper limb reaching task. They used a decision-making algorithm to automatically modify exercise parameters, which account for different individuals' specific needs and abilities. They have also used these parameters to make appropriate decisions about the rehabilitation exercises.

Another common understanding of adaptive rehabilitation mainly used in robotic and haptic systems is to actively adapt the assistance provided to the patient by a robot. This assistance is provided as per the physical needs of the patient. Wolbrecht et al. (2008) researched to examine different hypotheses on how to maximize the participation of the motor system through robotic assistance. Their findings reveal that a minimally assistive intervention previously introduced by Cai et al. (2006) termed as “assist-as-needed” is an appropriate strategy that can be used as the core for many assistive robots. Surprisingly, the “assist-as-needed” strategy coincides with a motor learning principle realized by Hasson et al. (2012), explaining that a human evolves his/her motor skills by minimizing the required force to control dynamically complex objects. A hypothesis is that the way an experienced therapist assists a motor-impaired patient is very similar to how the patient deals with high dynamical complexity objects. Thereby, the “assist-as-needed” strategy is comparable to traditional therapies. This can be the main reason underlying the effectiveness of assistive robots working based on the “assist-as-needed” strategy. Krebs and Hogan (2012) has mentioned that robotic therapy (RT) is reaching its tipping point and that RT practices, particularly based on motor learning principles, such as the “assist-as-needed” strategy, have been successful.

Most recently, Heuer and Lüttgen (2015) considered the assistive control strategies that work toward or against the motor recovery across trajectory and transformation learning skills. Their survey is accompanied by a classification of clinical results obtained from different strategies in terms of their effectiveness toward gaining certain motor skills. Maciejasz et al. (2014) adopted assistive control as one of the main high-level strategies of robotic therapy and added three more: challenge-based control, haptic stimulation, and couching control. To consolidate, Table 1 provides an overview of different adaptive rehabilitation approaches.

**Table 1:** Different adaptive rehabilitation approaches using virtual reality (VR) and robots.

**Publication**	**Adaptive rehabilitation technique**	**Modalities used for adaptation**		**Rehabilitation interface**	**Upper (U)/Lower (L) body**
		**Feedback from human**	**Feedback to human**		
Hocine et al. (2015)	Parameter adjustment	Estimation of task performance in terms of success rate of task completion Evaluation of subject's abilities in terms of maximum zone of 2D movements	Dynamic adjustment of task difficulty w.r.t. subject's ability and performance	Virtual Reality	U
Pehlivan et al. (2015)	Assistive control AAN (Assist-as-needed)	Subject performance	Modification of permissible error and assistance during movement execution	Robot	U
Perez-Ibarra et al. (2015)	Assistive control + Parameter adjustment	Estimation of force contribution and task performance using dynamic and kinematic feedback	Adjustment of level of assistance as well as the stiffness of impedance control	Robot	L
Squeri et al. (2014)	Assistive control	Subject's ability to keep up with target oscillations	Assistance adapted to residual capacities of motion while avoiding over-assistance	Robot	U
Barzilay and Wolf (2013)	Autonomous intervention planning	Estimation of task performance inferred by a trained neural network from biofeedback (EMG and Kinematic Info)	Planning rehabilitation tasks w.r.t. expectations of clinicians and feedbacks inferred from human	Virtual reality (Serious Games)	U
Pirovano et al. (2012)	Parameter adjustment	Estimation of task performance inferred by a fuzzy engine based on patients actions and Therapist's knowledge	Adjustment of task parameters, such as speed and range of motions+ Visual and voice effects are generated via an animated virtual therapist	Virtual reality (serious games)	U and L
Nirme et al. (2011)	Parameter adjustment	Estimation of the user model based on different parameters of task performance	Adjustment of task difficulty w.r.t. the estimated user model	Virtual Reality	U
Duff et al. (2010)	Autonomous intervention planning + Parameter adjustment	Estimation of task performance and recovery progress using kinematic feedback	Visual and musical stimulation are adapted by clinicians	Virtual reality (reaching task)	U

## 6. Discussion

**Figure 5:**
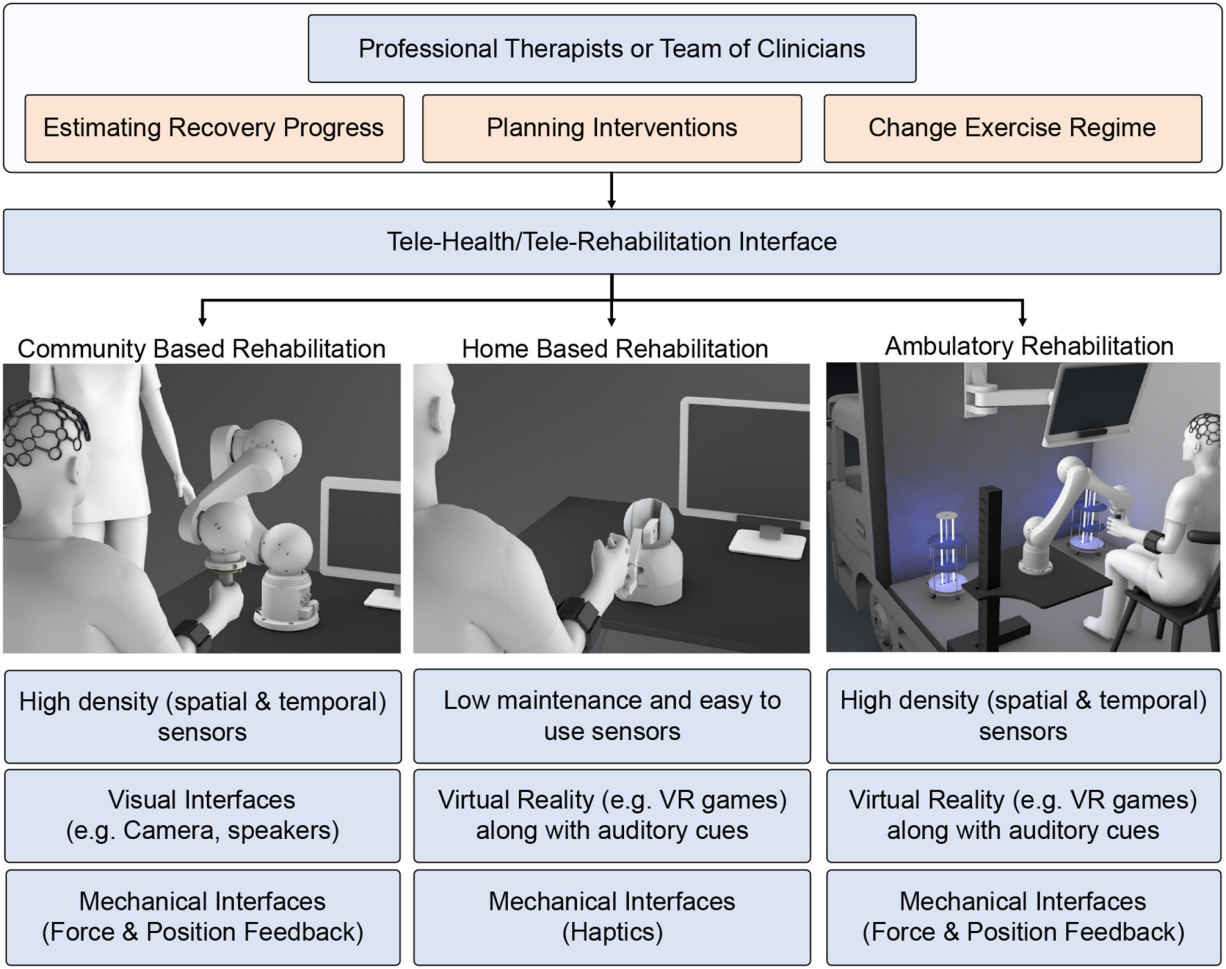
Schematic showing the rehabilitation therapy in three different setups: (1) community-based, (2) home-based, and (3) ambulatory. Present robotic systems are geared toward hospital or home-based approaches. However, due to COVID pandemic, we need to modify and adapt the current system taking into account social distancing norms, emotional stress due to lockdown, and safety of health care workers and patients.

In our opinion, the telerehabilitation procedure can serve as a safe and effective medium to continue the rehabilitation process while adhering to the safety guidelines during the COVID-19 outbreak. In teleoperated systems, patients and therapists interact through web-interfaces, and the clinical team can remotely monitor the progress of the patient and tune the system's parameters accordingly. Advances in robotic research have facilitated haptic devices that can sense the environment and adapt to it. Consequently, these devices can be used to collect the patient's information and provide necessary feedback effectively. This promotes lesser intervention from a clinical entity during the training process. Tele-rehabilitation can be conducted in-home, community, and as an ambulatory service catering to the needs of the patients (Figure 5).

Home-based rehabilitation has attracted many research studies in recent years due to its cost effectiveness and reliability. Moreover, patients can use it without any additional clinical assistance. Most of the therapy is concentrated in home environments to reduce the traffic toward clinical facilities. The therapists can provide rehabilitation instructions synchronously (real-time feedback) or asynchronously (exercise regime evaluated periodically). For such a setup, the basic requirements are a mobile phone and an Internet connection. Other simple sensors like EMG, IMU, and Kinect can also collect physiological signals for assessment. However, the haptic therapy outcome need not be superior unless the patient is actively engaged in the therapy. In this context, VR acts as a successful medium to promote active patient participation. VR can be hosted in-home environment easily without requiring any expensive setup. The therapy can use VR to deliver game-based exercises that are highly engaging for the patients and provide emotional support. Under certain circumstances, the patients must participate in high-intensity therapy sessions that the home-based setups cannot provide.

Intensive robotic therapy sessions can be easily accessed by the patients in community-based robotic rehabilitation centers. Community-based centers can accommodate heavy and expensive robots for intensive therapy. Patients can access these rehabilitation setups following social distancing guidelines and under a clinician or a volunteer's supervision. Any of the accessed systems can be sanitized and kept ready for the next patient. When patients access these community centers, clinicians can record necessary physiological information, such as EEG to examine patients' cognitive state otherwise not feasible in home-based environments. This allows for high-level monitoring and the metrics calculated through telerehabilitation services. For patients who have travel difficulties, the community-based rehabilitation can be extended as an ambulatory vehicle service with an onboard therapist to emulate the similar intensive robotic therapy experience provided in the community-based robotic therapy. The ambulatory vehicle can be equipped with high-end assistive devices (robotic) along with high-density physiological sensors (EEG, EMG) to assess the patient's state. In terms of health-guidelines, the ambulatory vehicle can be sanitized using UV light between two consequent therapy sessions. These vehicles can be accessed periodically to assess the patient's functional state better and simultaneously deliver high-intensity exercises using the equipped larger bandwidth robotic devices.

However, most of the robotic systems are not adaptive as they do not directly record feedback from the subjects or assess the patient's state. Hence, they require the intervention of clinical teams or doctors who can assess the improvements in the patients' functional state, which is difficult during the COVID-19 outbreak. Thus, there is a need for an adaptive strategy that synergistically combines humans' intentions and robots' dynamics; inevitably, VR is a great resource for such applications. With recent advances in user experience, VR and AR technology had provided an immersive environment during rehabilitation. This approach increases the patients' willingness to take part in the rehabilitation process, thus speeding up the recovery. Adaptive rehabilitation provides required assistance as needed and is a chief strategy underlying successful robotic rehabilitation. Such an adaptive robotic rehabilitation framework should assess and consider the patient's state as one of the main factors for providing appropriate assistance. In this direction, physiological signals have attracted a lot of attention as a reliable modality to assess the patient's state. The physiological signals, such as EEG, EMG, and eye-tracking can be used passively or actively to understand the patients' condition and modify rehabilitation parameters.

## 7. Conclusion

This paper presented a brief review of different telerehabilitation services that can be effectively used during the COVID-19 or similar pandemic and can serve as a reliable alternative to physical therapy. However, the rehabilitation service will be successful if the patients adhere to the routine and are engaged with the exercise regime. For this purpose, telerehabilitation should consider a reliable and cost-effective approach to measure the patient's engagement. Finally, as the therapists cannot deliver one-on-one therapy to patients due to the threat of spreading the virus, an adaptive rehabilitation setup is required with minimal intervention to deliver quality remote care and simultaneously assess the patient's progress.

## Author Contributions

All authors listed have made a substantial, direct and intellectual contribution to the work, and approved it for publication.

## Conflict of Interest

The authors declare that the research was conducted in the absence of any commercial or financial relationships that could be construed as a potential conflict of interest.
